# Structure of an engineered multidrug transporter MdfA reveals the molecular basis for substrate recognition

**DOI:** 10.1038/s42003-019-0446-y

**Published:** 2019-06-17

**Authors:** Hsin-Hui Wu, Jindrich Symersky, Min Lu

**Affiliations:** Department of Biochemistry and Molecular Biology, Rosalind Franklin University of Medicine and Science, 3333 Green Bay Road, North Chicago, IL 60064 USA

**Keywords:** Biochemistry, Structural biology

## Abstract

MdfA is a prototypical H^+^-coupled multidrug transporter that is characterized by extraordinarily broad substrate specificity. The involvement of specific H-bonds in MdfA-drug interactions and the simplicity of altering the substrate specificity of MdfA contradict the promiscuous nature of multidrug recognition, presenting a baffling conundrum. Here we show the X-ray structures of MdfA variant I239T/G354E in complexes with three electrically different ligands, determined at resolutions up to 2.2 Å. Our structures reveal that I239T/G354E interacts with these compounds differently from MdfA and that I239T/G354E possesses two discrete, non-overlapping substrate-binding sites. Our results shed new light on the molecular design of multidrug-binding and protonation sites and highlight the importance of often-neglected, long-range charge-charge interactions in multidrug recognition. Beyond helping to solve the ostensible conundrum of multidrug recognition, our findings suggest the mechanistic difference between substrate and inhibitor for any H^+^-dependent multidrug transporter, which may open new vistas on curtailing efflux-mediated multidrug resistance.

## Abstract (web summary)

Hsin-Hui Wu et al. present the structure of the I239T/G354E variant of the MdfA multidrug transporter in complex with three different ligands. They find that this variant has two discrete, non-overlapping substrate binding sites, leading to differential interactions with ligands compared to wild-type MdfA.

## Introduction

The inexorable rise of multidrug resistance undermines the efficacy of existing and new therapeutics and foreshadows a public health crisis^1^. The study of multidrug-resistance mechanisms and the development of novel therapeutic strategies to overcome multidrug resistance are therefore of prime importance and great urgency^2^. A principal mechanism underpinning the unabated multidrug resistance is mediated by integral membrane proteins known as multidrug transporters^1,3^. Thus far at least seven families of multidrug transporters have been identified: the ABC (ATP-binding cassette), the AbgT (*p*-aminobenzoyl-glutamate transporter), the DMT (drug/metabolite transporter), the MATE (multidrug and toxic compound extrusion), the MFS (major facilitator superfamily), the PACE (proteobacterial antimicrobial compound efflux), and the RND (resistance-nodulation-division) families^3^. The majority of the known multidrug transporters utilize the inwardly directed H^+^ electrochemical gradient to expel drugs through cellular membranes.

With >15,000 sequenced members, MFS is by far the largest solute transporter family^4–6^ and MdfA is one of the best characterized MFS multidrug transporters^7,8^. MdfA exhibits an extremely broad spectrum of drug recognition and can couple the export of cationic, neutral, and zwitterionic compounds to the import of H^+^, with a drug/H^+^ stoichiometry of 1:1 (refs. ^9–11^). The membrane-embedded D34 in MdfA appears to serve as the protonation site^11^. Moreover, previous studies identified a number of MdfA mutants with intriguingly different transport properties from those of the wild type protein^12–18^, including I239T/G354E. In sharp contrast to MdfA, which cannot extrude short dicationic compounds such as the neurotoxicant methyl viologen (MV)^19^, I239T/G354E can export both monocationic and short dicationic drugs, besides other well-known MdfA substrates^17^. Strikingly, biochemical studies suggested that the drug/H^+^ stoichiometry for I239T/G354E is 1:2 or 2:2, with the protein extruding one short dicationic or two monocationic drugs per transport cycle^17^. Notably, MdfA can also interact with two substrates simultaneously, although one inhibits the transport of the other^20^.

The structural characterization of MdfA lagged behind the functional study until recently, when the structures of MdfA and its mutants Q131R and Q131R/L339E were reported^21–24^. Among these structures are those of the inward-facing Q131R in complexes with zwitterionic n-dodecyl-n,n-dimethylamine-n-oxide (LDAO), monoanionic deoxycholate (DXC), and electroneutral substrate chloramphenicol. The chloramphenicol-binding site is located within the central cavity formed between the N and C domains, implying that the two domains rotate relative to each other around the chloramphenicol-binding site during transport, thereby alternately exposing the substrate- and H^+^-binding sites to the cytoplasm and periplasm^21^. This alternating access mechanism has gained experimental support from the outward-facing structure of MdfA^24^. Furthermore, the chloramphenicol-bound structure of Q131R suggests that chloramphenicol triggers the unbinding of H^+^ from the inward-facing transporter through direct H-bonding interactions with the side-chain carboxylate of D34.

Despite such advance, a number of critical issues regarding multidrug recognition remain unaddressed. Firstly, it is generally considered that multidrug transporters rarely interact with substrates through direct H-bonds, since these bonds are of specific lengths and directions, which place substantial limitations on the physicochemical property of substrates and are major contributors to the selectivity of molecular interaction^1,7^. As such, the functionally important H-bonds observed between the substrate (chloramphenicol) and Q131R (D34 and N33) seem to belie the promiscuous nature of multidrug recognition, begging the question of how MdfA achieves its substrate polyspecificity^8^. Secondly, it takes only a minor change to alter the substrate specificity and drug/H^+^ stoichiometry of MdfA, since the double mutation I239T/G354E enabled the transporter to recognize and extrude one short dicationic or two monocationic drugs simultaneously^17^. Without any structural information on such mutant, however, it is largely unknown how the already broad substrate specificity of MdfA can still be expanded, and whether I239T and G354E serve as drug-binding residues and/or protonation sites.

To answer these questions, we report the crystal structures of I239T/G354E in complexes with LDAO, MV and DXC (Supplementary Fig. 1), determined at resolutions up to 2.2 Å. Perhaps surprisingly, our data support two discrete, non-overlapping LDAO-binding sites within I239T/G354E, which helps to explain how this mutant exports two drugs simultaneously. Furthermore, we found that the I239T/G354E mutation both broadened (for MV) and narrowed (for DXC) the drug recognition spectrum of MdfA, largely by altering the transporter-drug interactions. Combining structural and biochemical studies, our work provides a conceptual framework for understanding the mechanistic differences between MdfA and I239T/G354E. Our findings also prompt a re-evaluation of the existing dogmas of molecular recognition and may open new prospects for curbing efflux-mediated multidrug resistance.

## Results

### The LDAO-bound structure of I239T/G354E

The best diffracting crystals of I239T/G354E were obtained in the presence of LDAO at pH 8. The resulting structure was determined to 2.2 Å resolution by combining molecular replacement and single wavelength anomalous dispersion (SAD) phasing (Table 1). The experimental SAD phases were valuable in several regards. Firstly, they allowed us to objectively examine the potential structural differences, if any, between I239T/G354E and Q131R. Secondly, they yielded bias-free electron density maps that revealed the locations of the bound ligands (see below). Thirdly, they increased data to parameter ratios during structure refinement and improved the model quality.

**Table 1:** Data collection and structure refinement statistics

	LDAO-bound I239T/G354E	MV-bound I239T/G354E	DXC-bound I239T/G354E
*Data collection*			
Space group	C2	C2	C2
Cell dimensions			
a,b,c (Å)	95.24, 63.03, 102.34	95.07, 63.15, 102.07	93.48, 71.37, 113.14
α,β,γ (°)	90, 101.28, 90	90, 100.87, 90	90, 109.73, 90
Resolution (Å)	100.0–2.2 Å	100.0–2.8 Å	100.0–3.0 Å
R_sym_^a^	0.08 (0.66)	0.07 (0.41)	0.04 (0.54)
CC_1/2_^b^	0.99 (0.26)	0.99 (0.68)	0.99 (0.27)
I/σ	9.5 (1.0)	12.6 (1.4)	40.0 (1.1)
Completeness (%)	92.5 (53.9)	90.4 (86.5)	98.0 (76.4)
Redundancy	2.7	4.7	14.9
*Phasing*			
Resolution range	20.0–3.0 Å	20.0–3.5 Å	20.0–4.0 Å
Phasing power^c^	1.01	1.13	1.24
R_cullis_^d^	0.99	0.97	0.93
Figure of merit^e^	0.23	0.27	0.34
*Refinement*			
Resolution range	15.0–2.2 Å	15.0–2.8 Å	15.0–3.0 Å
No. reflections	26746	12656	10817
R_cryst_^f^/R_free_^g^ (%)	22.2/24.8	20.4/24.2	26.9/29.3
No. atoms	3082	3007	2951
<B>_protein_	49	60	100
<B>_ligand_	50	65	98
<B>_water_	60	57	N.A.
r.m.s. deviations			
Bond lengths (Å)	0.006	0.005	0.009
Bond angles (^o^)	1.0	1.0	1.2
Ramachandran			
favored	99.7%	100%	99.4%
allowed	0.3%	0%	0.6%
disallowed	0%	0%	0%

^a^R_sym_  =  Σ| I−<I> | /ΣI, where I is the observed intensity of symmetry-related reflections

^b^CC_1/2_ is the half-split Pearson correlation coefficient

^c^Phasing power  =  F_h_ / E, where F_h_ is the rms isomorphous/anomalous difference and E the rms residual lack-of-closure

^d^R_cullis_ (ano) = Σ(||ΔFPH(obs)|−|ΔFPH(calc)||) / Σ|ΔFPH(obs)|, where ΔFPH(obs) and ΔFPH(calc) are the observed and calculated structure factor differences between Bijvoet pairs, respectively

^e^Figure of merit is defined as weighted mean value of the cosine of phase error

^f^R_cryst_  = Σ(||F_obs_|−|F_calc_||) / Σ(|F_obs_|), where F_obs_ and F_calc_ are the observed and calculated structure factors, respectively

^g^R_free_ is the same as R_cryst_ but calculated with 5% of the reflections excluded from structure refinement

**Fig. 1:**
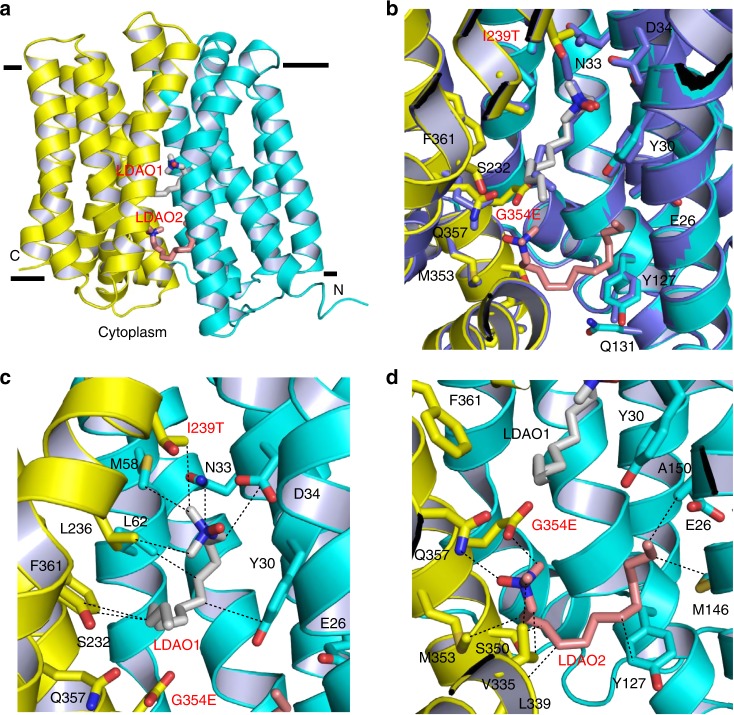
Structure of the LDAO-bound I239T/G354E. **a** Structure of I239T/G354E as viewed from the membrane. I239T/G354E is drawn as a ribbon diagram, with the N (9–205) and C (206–400) domains colored cyan and yellow, respectively. The bound LDAO molecules are shown as stick models and are colored grey and light pink, respectively. **b** Overlay of the LDAO-bound structures of Q131R (light blue, PDB 4ZP2) and I239T/G354E, with the LDAO-binding amino acids and LDAO drawn as stick models. The Q131R-bound LDAO is colored light blue. Q131R was modeled as alanine in 4ZP2; in I239T/G354E, Q131 makes no contact with the bound LDAO molecules. **c** Close-up view of the LDAO1-binding site, LDAO1 and the relevant amino acids are shown in stick models and close-range interactions are highlighted as dashed lines. **d** Structure of the LDAO2-binding site, LDAO2 and the relevant amino acids are drawn as sticks, dashed lines indicate the interactions between LDAO2 and I239T/G354E

Overall, the LDAO-bound structure of I239T/G354E (Fig. 1a) is similar to that of Q131R, which could be superimposed onto each other to yield a rms deviation of 1.0 Å for 390 common Cα atoms (Fig. 1b). This similarity suggested that our structure captures the transporter in an inward- or cytoplasm-facing state, and that the respective mutation gave rise to no global structural change even though I239T/G354E or Q131R reportedly altered the transport function^17,23^. Perhaps unexpectedly, the density-modified SAD maps revealed two continuous, unbranched non-protein electron density features within I239T/G354E, indicating two bound LDAO molecules, denoted LDAO1 and LDAO2 (Supplementary Fig. 2). LDAO1, similar to that seen in the LDAO-bound Q131R structure (Fig. 1b), is seated halfway into the lipid bilayer, at roughly equal distance between the extracellular and intracellular surfaces of the membrane (Fig. 1a). LDAO2, by contrast, is situated between LDAO1 and the cytoplasm-membrane interface, ~6 Å from the latter and appreciably more solvent-accessible than LDAO1. The closest approach of LDAO1 and LDAO2 exceeds 5 Å, implying that they interact with the transporter independently. Moreover, the structures of LDAO-binding sites in MdfA and I239T/G354E are similar (see below), implying that the binding of one LDAO molecule is unlikely to allosterically affect the binding of the other by altering the protein structures. Of note, previous studies suggested that the binding of two substrate molecules to I239T/G354E is not cooperative^17^.

The polar head of LDAO1 engages in van der Waals interactions with the side chains of M58, L236 and I239T (Fig. 1c). The positively charged N1 of LDAO1 is 3.7 Å away from the side-chain carboxylate in D34 (Supplementary Table 1), likely forming a long-range charge-charge interaction^25–27^. The aliphatic chain of LDAO1, on the other hand, makes contacts with the side chains of Y30, N33, D34, L62, S232 and F361. The interactions between LDAO1 and I239T/G354E closely resemble those between LDAO and Q131R (Fig. 1b), implying that the LDAO-binding site in MdfA is preserved in I239T/G354E. By contrast, the binding site of LDAO2 was previously uncharacterized^21^. Specifically, the polar head of LDAO2 makes van der Waals interactions with the side chains of V335 and G354E (Fig. 1d). The negatively charged O1 of LDAO2 forms an H-bond with the side-chain amide of Q357, and the positively charged N1 of LDAO2 is 5.3 Å from the side-chain carboxylate of G354E, likely making another long-range electrostatic interaction. Additionally, the aliphatic chain of LDAO2 makes contacts with the side chains of Y127, M146, A150, L339, S350, and M353 through van der Waals interactions. Notably, although some water molecules were resolved in the I239T/G354E structure, none of them seems to mediate the interaction between I239T/G354E and LDAO1 or LDAO2.

The structural comparison between the LDAO2-binding amino acids in I239T/G354E and their counterparts in Q131R (Fig. 1b) suggested that the binding of LDAO2 and I239T/G354E mutation led to virtually no change in the protein structure, with the side chains of only Y30, Y127 and Q357 making small but noticeable changes. Apparently, the addition of the side chain carboxylate of G354E, rather than substantial protein structural change, enables I239T/G354E to interact with a second LDAO molecule. Although D34 and G354E are both located within the multidrug-binding cavity^21^, their side-chain carboxylates are 12.8 Å apart. This distance appears to allow I239T/G354E to bind two LDAO molecules simultaneously since the two binding sites are well-separated and hence spatially feasible. It is conceivable that if D34 and G354E were located too close toward each other, they would give rise to unfavorable electrostatic repulsion to destabilize the protein, and the binding of LDAO1 and LDAO2 would be mutually exclusive.

### Functional importance of the LDAO-binding sites

**Fig. 2:**
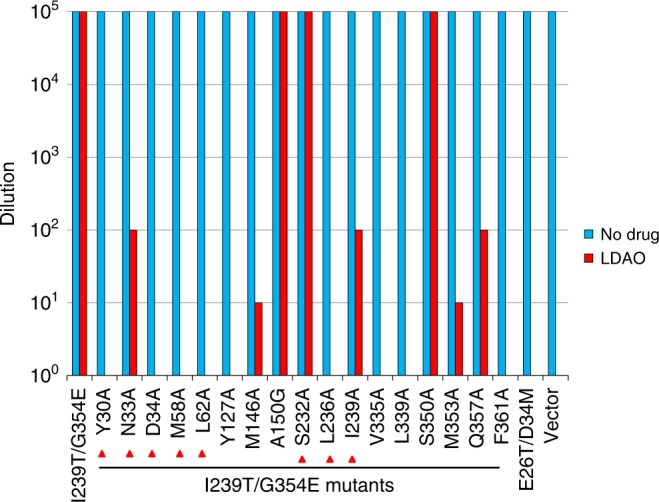
LDAO resistance assay of I239T/G354E variants. Bacteria expressing the I239T/G354E variants were tested for LDAO resistance in solid media. Five consecutive 10-fold dilutions of bacteria were prepared, and 4 µL of each dilution were plated on plates containing kanamycin, IPTG and 0.01% LDAO. The ability of bacteria to form single colonies was visualized after overnight incubation. The height of the bars corresponds to the maximal dilution at which bacterial growth was detected. The experiments were repeated >3 times. Mutations of the amino acids that interact with LDAO1 in I239T/G354E were indicated by red triangles

To probe the functional relevance of the observed protein-LDAO interactions, we first asked if LDAO is a substrate for MdfA or I239T/G354E. We found that the expression of MdfA or I239T/G354E in *E. coli* rendered the bacterium resistant to the cytotoxic effects of LDAO, suggesting that both MdfA and I239T/G354E can extrude LDAO (Supplementary Fig. 3 and Fig. 2). We then mutated some of the LDAO-interacting amino acids identified from the Q131R structure (Supplementary Fig. 4), and tested the function of these single mutants in the LDAO susceptibility assay. Bacteria expressing both the vector and E26T/D34M, an inactive MdfA mutant^11^, were used to measure the background level of cellular resistance to LDAO, which suggested that the endogenous efflux transporters exerted negligible effect on the resistance assay (Supplementary Fig. 3). Moreover, we found that the mutations of Y30, N33, D34, M58, and L236 abrogated the ability of MdfA to confer LDAO resistance to *E. coli*. By contrast, the mutations of L62, S232, and Q357 had little impact on the transport function. Notably, Q357 is removed from the LDAO-binding site in Q131R. Overall, our data demonstrated the functional importance of Y30, N33, D34, M58, and L236 in the MdfA-mediated export of LDAO.

Additionally, we replaced the LDAO-binding amino acids in I239T/G354E individually with alanine or glycine (for A150), and then examined the mutational effects in the LDAO resistance assay (Fig. 2). We observed that mutations of Y30, D34, M58, L62, Y127, L236, V335, L339, and F361 to alanine abolished the ability of I239T/G354E to confer LDAO resistance to *E. coli*. Moreover, alanine substitutions of N33, M146, I239T, M353 and Q357 impaired the transport function of I239T/G354E, albeit to a lesser extent. By stark contrast, mutations of A150, S232 and S350 exerted little deleterious effect on the transport function. To exclude the possibility that the Y30A, D34A, M58A, L62A, Y127A, L236A, V335A, L339A, or F361A mutation abrogated the transport function of I239T/G354E by disrupting the protein folding, we analyzed these mutants by using gel filtration chromatography (Supplementary Fig. 5). We observed that these detergent-purified mutants all eluted as sharp and symmetrical peaks and therefore are well-folded^28,29^. Taken together, our data strongly suggested that the interactions between I239T/G354E and LDAO1/LDAO2 seen in our structure are functionally important, and that Y30, N33, D34, M58, L62, Y127, M146, L236, I239T, V335, L339, M353, Q357, and F361 play pivotal roles in the I239T/G354E-mediated efflux of LDAO.

**Fig. 3:**
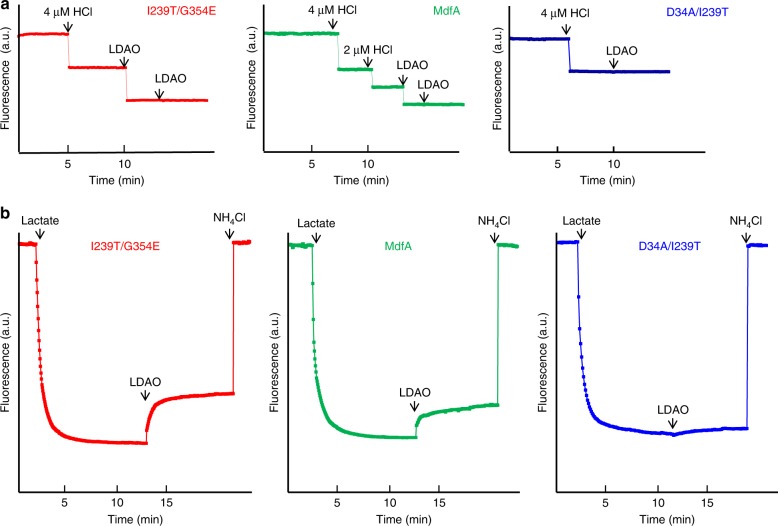
Functional characterization of I239T/G354E. **a** Fluorescence measurement of a solution containing 2 µM I239T/G354E (red) or MdfA (green), revealing its ability to release two or one proton per protein molecule upon LDAO binding. As a comparison, the addition of LDAO to a solution containing 2 µM D34A/I239T (blue), failed to trigger the release of H^+^. **b** LDAO/H^+^ antiport observed in the everted membrane vesicles expressing I239T/G354E (red) or MdfA (green). H^+^ movement was monitored by measurement of acridine orange fluorescence, which is shown in arbitrary units (a.u.). By contrast, LDAO failed to trigger H^+^ movement in the everted membrane vesicles harboring D34A/I239T (blue). The traces are representative of experiments performed in triplicate using two different preparations of everted membrane vesicles

Our data also implied that I239T/G354E binds and exports two LDAO molecules simultaneously, most probably with LDAO1 and LDAO2 triggering the release of H^+^ from the side-chain carboxylate of D34 and G354E, respectively. In accord with this mechanism, the binding of LDAO to the purified I239T/G354E induced the dissociation of two protons from the transporter (Fig. 3a). By contrast, the addition of LDAO to a solution containing MdfA, which lacks G354E, or D34A/I239T/G354E, which lacks D34, triggered the release of only one proton from the protein (Fig. 3a and Supplementary Fig. 6). Furthermore, the binding of LDAO to purified D34A/I239T, which lacks both D34 and G354E, failed to trigger any H^+^ release (Fig. 3a). To reaffirm the important roles of D34 and G354E in the export of LDAO, we used the everted (inside out) membrane vesicles to study the H^+^/LDAO antiport. We found that LDAO elicited the counter-movement of H^+^ in everted membrane vesicles expressing I239T/G354E and MdfA, but not D34A/I239T/G354E, D34A/I239T or vector (Fig. 3b and Supplementary Fig. 6). These observations suggested that I239T/G354E or MdfA mediated the LDAO-induced movement of H^+^ in membrane vesicles, which is unlikely caused by the endogenous efflux transporters. Taken together, our data supported that I239T/G354E catalyzes the exchange of LDAO1/LDAO2 for two protons per transport cycle, with D34 and G354E serving as the protonation sites.

Given the pH of the crystal solution (~8) and the measured^11,17^ and calculated^30^ pK_a_ for D34 and G354E (~7), our structure likely represents a LDAO-bound, deprotonated I239T/G354E. Our findings thus gave new insights into how I239T/G354E exports two drugs simultaneously, a characteristic that may be shared among some multidrug transporters^31–33^. Importantly, our data implied that the two substrate-binding sites in I239T/G354E are functionally non-equivalent: the transporter (MdfA) with an intact LDAO1-binding site remains functional despite a lack of G354E (Fig. 3b and Supplementary Fig. 6), whereas the transporter (D34A/I239T/G354E) with an intact LDAO2-binding site is inactive once D34 is mutated to alanine. Moreover, previous studies showed that MdfA extruded monocationic compounds, but the mutant D34M/I239T/G354E could not^17^. All these data suggested that the centrally located D34 plays a more important role than the peripherally located G354E. One plausible explanation for this difference is that the binding of substrate and/or H^+^ to D34 triggers the protein conformational changes required for transport more effectively than that of G354E.

### The MV-bound structure of I239T/G354E

**Fig. 4:**
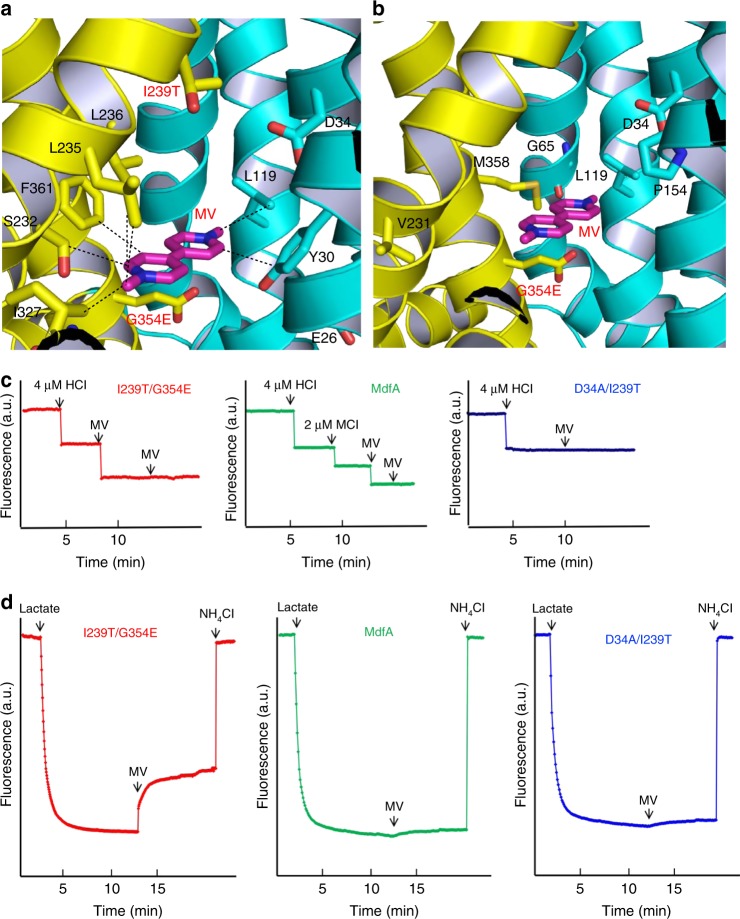
Recognition and extrusion of MV by I239T/G354E. **a** Close-up view of the MV-binding site in I239T/G354E, with the N and C domains colored cyan and yellow, respectively. The bound MV is drawn in stick models and colored magenta. The relevant amino acids are shown in stick models and close-range interactions are highlighted by dashed lines. **b** Location of the mutated amino acids that resulted in the new functionality of transporting short dicationic compounds. The bound MV (magenta) and relevant amino acids are shown in stick models. **c** Fluorescence measurement of a solution containing 2 µM I239T/G354E (red) or MdfA (green), revealing its ability to release two or one proton per protein molecule upon MV binding. As a comparison, the addition of MV to a solution containing 2 µM D34A/I239T (blue), failed to trigger the release of H^+^. **d** MV/H^+^ antiport observed in the everted membrane vesicles expressing I239T/G354E (red). H^+^ movement was monitored by measurement of acridine orange fluorescence, which is shown in arbitrary units (a.u.). By contrast, MV failed to trigger H^+^ movement in the everted membrane vesicles harboring MdfA (green) or D34A/I239T (blue). The traces are representative of experiments performed in triplicate using two different preparations of everted membrane vesicles

To uncover how the I239T/G354E mutation enabled MdfA to recognize short dicationic drugs^17^, we determined the 2.8 Å structure of I239T/G354E bound to MV, by combining molecular replacement and SAD phasing (Table 1). The resulting structure is similar to that of LDAO-bound I239T/G354E, with a rms deviation of 0.5 Å over 392 Cα positions. The experimental maps revealed conspicuously flat, insole-shaped electron density for the bound MV, anchored at the apex of the cytoplasm-facing cavity (Supplementary Fig. 7). The bound MV resides about halfway into the membrane and makes numerous contacts with the transporter, mostly via hydrophobic interactions (Fig. 4a). In particular, the bound MV engages in van der Waals interactions with the side chains of Y30, L119, S232, L235, L236, I327, and Y361 in I239T/G354E (Supplementary Table 1). Furthermore, the side-chain carboxylate groups of D34 and G354E are 8.3 and 5.8 Å away from the positively charged N2 in MV, respectively (Fig. 4b). As the positive charges in MV are delocalized, D34 and G354E likely make long-range electrostatic interactions with MV, with the closest approach being 7.3 and 5.2 Å, respectively. Additionally, the side-chain carboxylate in E26, 7.9 Å away from the positively charged N2 in MV, may also contribute to the neutralization of this dicationic substrate. Notably, these long-range electrostatic interactions seem effective in mediating the MV-triggered deprotonation of the transporter (see below, Fig. 4c, d).

**Fig. 5:**
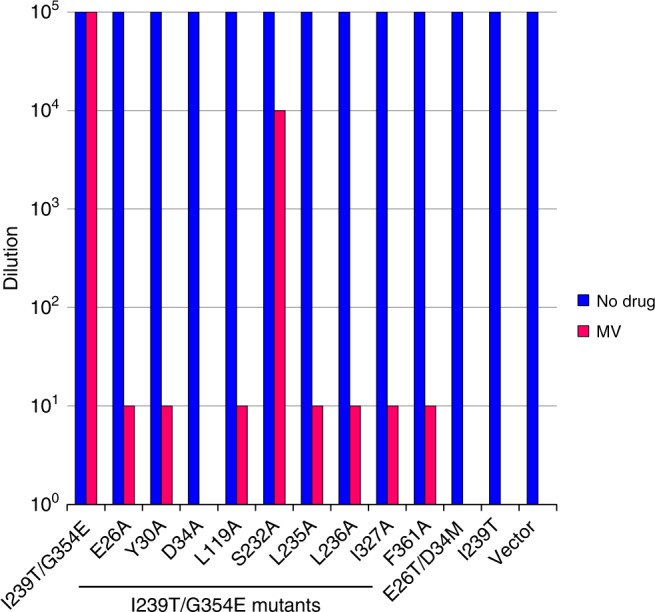
MV resistance assay of I239T/G354E variants. Bacteria expressing the I239T/G354E variants were tested for MV resistance in solid media. Five consecutive 10-fold dilutions of bacteria were prepared, and 4 µL of each dilution were plated on plates containing kanamycin, IPTG and 30 µg/mL MV. The ability of bacteria to form single colonies was visualized after overnight incubation. The height of the bars corresponds to the maximal dilution at which bacterial growth was observed. The experiments were repeated >3 times

To assess the functional importance of these interactions, we mutated E26, Y30, D34, L119, S232, L235, L236, I327, and F361 to alanine in I239T/G354E, and replaced I239 by threonine in MdfA, and then examined the ability of these mutants to confer resistance against MV to *E. coli* (Fig. 5). We observed that the alanine substitution of D34 abolished the ability of I239T/G354E to render bacteria resistant to MV, and the mutations of E26, Y30, L119, L235, L236, I327, and F361 to alanine impaired the ability of I239T/G354E to confer protection against MV. By contrast, the mutation of S232 had little adverse effect. Furthermore, the expression of I239T conferred no MV resistance to bacteria. Additionally, detergent-purified mutants E26A, Y30A, D34A, L119A, L235A, L236A, I327A, and F361A were found to be well-folded on the basis of their gel filtration profiles (Supplementary Fig. 5). Overall, our data implied that E26, Y30, D34, L119, L235, L236, I327, G354E, and F361 have important roles in the expulsion of MV by I239T/G354E.

**Fig. 6:**
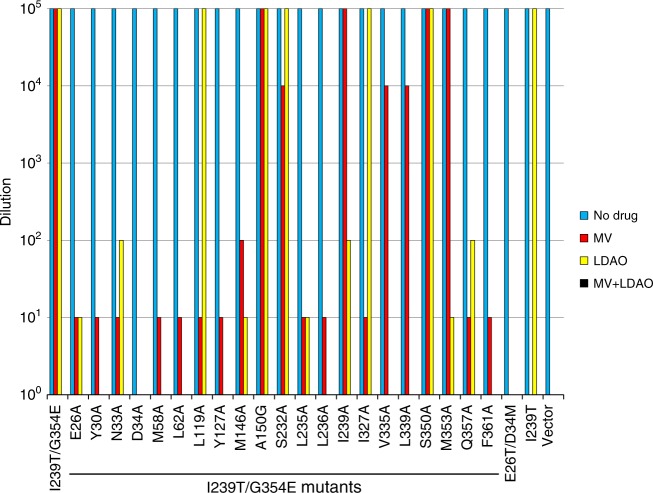
Drug resistance assay of I239T/G354E variants. Bacteria expressing the I239T/G354E variants were tested for MV and/or LDAO resistance in solid media. Five consecutive 10-fold dilutions of bacteria were prepared, and 4 µL of each dilution were plated on plates containing kanamycin, IPTG, 30 µg/mL MV or 0.01% LDAO, or in the presence of both 30 µg/mL MV and 0.01% LDAO. The ability of bacteria to form single colonies was visualized after overnight incubation. The height of the bars corresponds to the maximal dilution at which bacterial growth was detected. The experiments were repeated >3 times

Our structures of I239T/G354E also indicate that the binding site of MV overlaps with that of LDAO1, so we tested the ability of LDAO-binding site mutants to confer MV resistance (Fig. 6). We found that the mutations of Y30, N33, D34, M58, L62, Y127, M146, L236, Q357, and F361 markedly reduced the ability of I239T/G354E to confer resistance against MV, whereas the mutations of A150, S232, I239T, V335, L339, S350, and M353, most of which bind LDAO2, had little deleterious effect. These results dovetail with the observation that the LDAO2- and MV-binding sites are distinct and non-overlapping. Furthermore, we observed that LDAO enhanced the bactericidal activity of MV, probably by competing for the LDAO1-binding amino acids. After we examined the sensitivity of the substrate-binding mutants to LDAO in MV resistance assay, we found that LDAO reduced the ability of these mutants to confer cellular resistance against MV, regardless of whether the mutated residues bind LDAO1, LDAO2 or MV. Our data thus implied that LDAO competitively inhibits the export of MV by I239T/G354E, and vice versa.

Notably, prior studies suggested that I239T/G354E catalyzes the exchange of one MV for two protons during transport^17^. Our MV-bound structure of I239T/G354E revealed one MV bound to the transporter. Furthermore, we discovered that the binding of MV to the purified I239T/G354E triggered the stoichiometric release of two protons from the transporter (Fig. 4c), and MV also induced the counter-movement of H^+^ in everted membrane vesicles harboring I239T/G354E (Fig. 4d). By sharp contrast, MV triggered the release of only one proton from MdfA and D34A/I239T/G354E, and no H^+^ from purified D34A/I239T (Fig. 4c and Supplementary Fig. 8). Moreover, MV was unable to induce the H^+^ movement in the inside out membrane vesicles containing MdfA, D34A/I239T/G354E, D34A/I239T or vector (Fig. 4d and Supplementary Fig. 8). Altogether, our data suggested that MV induces the unbinding of H^+^ from D34 and G354E in I239T/G354E during transport. Since the bound MV is located >3 Å from the side-chain carboxylates in D34 and G354E, we argue that MV triggers the deprotonation of D34 and G354E through long-range electrostatic interactions.

Besides I239T/G354E, previous studies^17^ have identified MdfA single mutants G65E, L119E, P154E, V231E, G354E and M358E as capable of exporting short dicationic drugs such as MV. In our MV-bound structure, the side chains of G65, L119, P154, V231, G354E and M385 are 6.3, 3.8, 6.8, 6.7, 5.2 and 4.3 Å away from the bound ligand, respectively (Fig. 4b), implying that the side-chain carboxylates of those mutated residues interact with the dicationic substrate through long-range electrostatic interactions in the corresponding mutants. Notably, G65, L119, P154, V231, G354E and M358 surround the bound MV, and are positioned 13.0, 8.7, 3.8, 16.9, 13.9 and 12.1 Å away from D34, respectively (Fig. 4b). Since these mutated residues are within the multidrug-binding cavity that harbors many hydrophobic amino acids, the pKa of their side-chain carboxylates is likely shifted similarly to that of G354E, so that the mutated residues can serve as protonation sites in vivo^11^. Therefore, we contend that if the added acidic amino acid surrounds the substrate and is well-separated from D34 (≥4 Å), the resulting MdfA mutant may gain the ability of recognizing short dicationic drugs via long-distance electrostatic interactions, with the added amino acid acting as both substrate-binding residue and protonation site.

### The DXC-bound structure of I239T/G354E

**Fig. 7:**
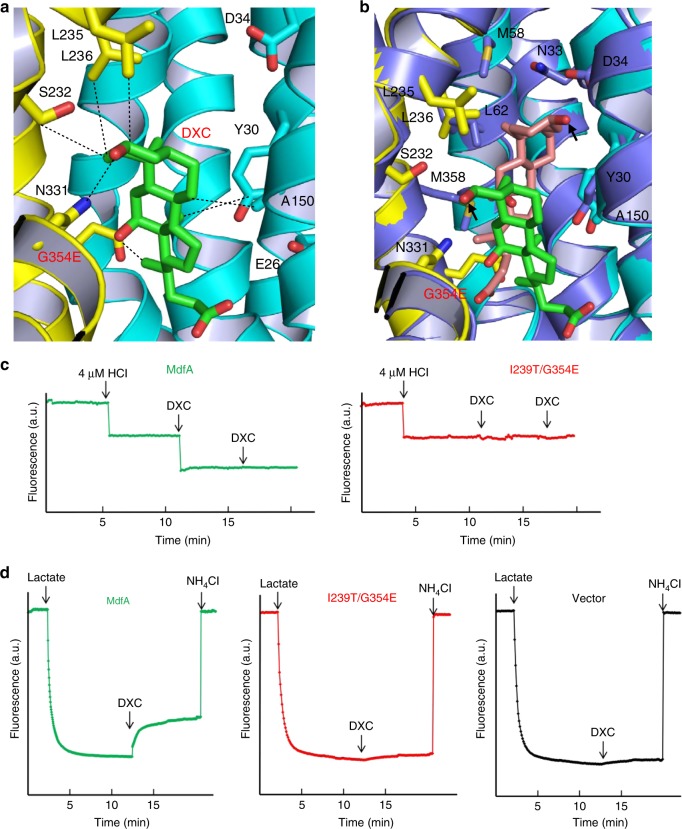
Interactions between I239T/G354E and DXC. **a** Close-up view of the DXC-binding site within I239T/G354E, with the N and C domains colored cyan and yellow, respectively. The bound DXC (green) and relevant amino acids are shown in stick models, and the close-range interactions are highlighted by dashed lines. **b** Overlay of the DXC-bound Q131R (light blue, PDB 4ZP0) and I239T/G354E, with the DXC-binding amino acids and DXC drawn as stick models. The Q131R-bound DXC is colored light pink and the C1-OH groups are highlighted by black arrows. **c** Fluorescence measurement of a solution containing 4 µM MdfA (green), revealing its ability to release one proton per protein molecule upon DXC binding. As a comparison, the addition of DXC to a solution containing 4 µM I239T/G354E (red), failed to trigger the release of H^+^. **d** DXC/H^+^ antiport observed in the everted membrane vesicles expressing MdfA (green). H^+^ movement was monitored by measurement of acridine orange fluorescence, which is shown in arbitrary units (a.u.). By contrast, DXC failed to trigger H^+^ movement in the everted membrane vesicles harboring I239T/G354E (red) or vector (black). The traces are representative of experiments performed in triplicate using two different preparations of everted membrane vesicles

As I239T/G354E interacts with zwitterionic and dicationic ligands differently from MdfA, we asked if this difference extends to anionic compounds such as DXC. Toward this goal, we determined the 3.0 Å structure of I239T/G354E bound to DXC by combining molecular replacement and SAD phasing (Table 1). The resulting DXC-bound structure of I239T/G354E is similar to that of Q131R, with an rms deviation of 1.0 Å for 380 equivalent Cα atoms. The experimental electron density maps revealed the bound DXC in the cytoplasm-facing cavity (Supplementary Fig. 9), which makes a number of close-range interactions with I239T/G354E. Specifically, DXC engages in van der Waals interactions with the side chains of Y30, A150, S232, L235, L236, and G354E, whereas the side chain of N331 forms an H-bond with the C1-OH from DXC (Fig. 7a).

Notably, the interactions between the DXC and I239T/G354E are markedly different from those between DXC and Q131R (Fig. 7b). If the DXC from our structure is overlaid onto its counterpart in the DXC-Q131R complex by superimposing the protein Cα backbones, the distance between the two C1-OH groups in DXC exceeds 8.6 Å. Apparently, the addition of side-chain carboxylate of G354E, rather than the change in protein conformation, altered the binding pose of DXC within the transporter. Notably, the C23-carboxylate group of DXC would be positioned too close to the side–chain carboxylate of G354E and cause unfavorable electrostatic repulsion, if I239T/G354E would maintain the same interactions with DXC as Q131R. Given the pH of the crystallization solution (~6), the DXC-bound structure of I239T/G354E likely portrays the protonated transporter bound to a protonated DXC.

We next asked if MdfA or I239T/G354E can extrude DXC. By utilizing the drug susceptibility assay, we found that the expression of MdfA or I239T, but not G354E or I239T/G354E, substantially enhanced the resistance of *E. coli* to DXC (Supplementary Fig. 10). Apparently, the mutation G354E abolished the ability of MdfA to extrude DXC. Furthermore, DXC induced the counter-movement of H^+^ in everted vesicles expressing MdfA, but not I239T/G354E (see below, Figs. 7c, d). Therefore, we concluded that DXC is a transportable substrate for MdfA, but not I239T/G354E. We then mutated the DXC-binding amino acids based on the DXC-bound Q131R structure (Fig. 7b), and tested the ability of the MdfA mutants to confer cellular resistance to DXC (Supplementary Fig. 10). We found that the expression of MdfA mutants N33A, D34A, M58A, L236A, or Q357A was unable to render *E. coli* resistant against DXC, implying that these amino acids play pivotal roles in the MdfA-mediated extrusion of DXC, and that the mutation G354E or I239T/G354E altered the substrate specificity of MdfA by precluding the protein from extruding DXC.

**Fig. 8:**
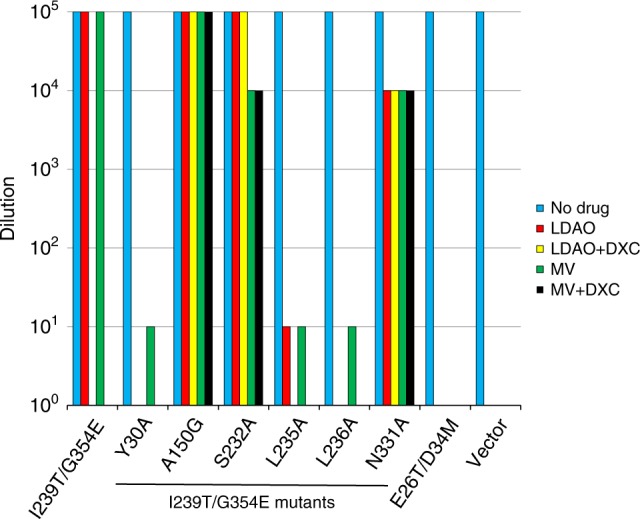
Drug resistance assay of I239T/G354E variants. Bacteria expressing the I239T/G354E variants were tested for drug resistance in solid media. Five consecutive 10-fold dilutions of bacteria were prepared, and 4 µL of each dilution were plated on plates containing kanamycin, IPTG and 0.01% LDAO, or 30 µg/ml MV, in the absence and presence of 300 µg/mL DXC. The ability of bacteria to form single colonies was visualized after overnight incubation. The height of the bars corresponds to the maximal dilution at which bacterial growth was detected. The experiments were repeated at least three times

Although DXC is not transportable by I239T/G354E, it potentiated the bactericidal activity of LDAO or MV (Fig. 8). Notably, the binding site of DXC overlaps with those of LDAO1, LDAO2 and MV. Therefore, DXC likely hinders the binding of LDAO or MV to the transporter as a competitive inhibitor. To study the importance of the interactions observed between DXC and I239T/G354E, we mutated the DXC-binding amino acids and tested their ability to confer cellular resistance against LDAO or MV, both in the absence and presence of DXC. In the absence of DXC, we found that the mutations of Y30, L235, and L236 abrogated the ability of I239T/G354E to confer cellular resistance to LDAO or MV. By stark contrast, the I239T/G354E mutants A150G, S232A, and N331A retained the transport function. In the presence of DXC, however, I239T/G354E was unable to confer cellular resistance to LDAO or MV, while the I239T/G354E mutants A150G, S232A, and N331A still rendered bacteria resistant to LDAO or MV. Our data thus indicated that the mutations of A150, S232 and N331 reduced the sensitivity to DXC, likely by weakening the binding of DXC to I239T/G354E.

Our structure helps to explain why DXC is a transportable substrate for MdfA, but not for I239T/G354E. Specifically, in Q131R, an H-bond is formed between the side-chain carboxylate of D34 and the C1-OH of DXC (Fig. 7b), which likely triggers the deprotonation of D34; in I239T/G354E, however, no such interaction is made between DXC and D34 or G354E (Fig. 7a). Thus, we expect the binding of DXC to trigger the release of H^+^ from MdfA, but not I239T/G354E. Congruently, the addition of DXC promoted a stoichiometric release of one H^+^ from the purified MdfA, but not I239T/G354E (Fig. 7c). Moreover, DXC evoked the counter movement of H^+^ in everted membrane vesicles expressing MdfA, but not I239T/G354E or vector (Fig. 7d). Altogether, our data suggested that MdfA catalyzes the exchange of DXC for one H^+^, whereas DXC acts as a non-transportable inhibitor for I239T/G354E.

## Discussion

This work presents the first, to our knowledge, structural characterization of any engineered multidrug antiporter with altered substrate specificity. Our data strongly suggested that MdfA interacts with electrically distinct substrates differently: it utilizes H-bonds to recognize electroneutral (such as chloramphenicol) and anionic substrates (such as DXC); whereas it recognizes zwitterionic (such as LDAO) and cationic substrates through charge-charge interactions. The broad substrate specificity of MdfA apparently arises from the preponderance of non-specific hydrophobic interactions made between the substrate and transporter, as well as the involvement of long-range charge-charge interactions. Furthermore, the existence of two discrete drug-binding sites, confirmed by the LDAO-bound I239T/G354E structure, can accommodate one or two drugs of various sizes and shapes, may also contribute to the broad substrate specificity of MdfA.

Notably, specific H-bonds are involved in the interactions between MdfA and chloramphenicol or DXC, which bears some resemblance to the substrate-specific MFS transporters that utilize H-bonding interactions to select substrate^34,35^. Consequently, any mutation that alters specific H-bonding interactions between MdfA and substrate, either by direct removal of the amino-acid side-chain involved in the H-bonds (D34A), or by indirectly affecting the orientation of the bound ligand (G354E), would change the substrate specificity, as exemplified by our DXC-bound structure (Fig. 7a). Moreover, our LDAO- and MV-bound structures suggested that zwitterionic and cationic substrates can be recognized through long-range charge-charge interactions (Figs. 1 and 4a). Such long-range interactions, which are likely enhanced >10-fold by the low-dielectric environment^25–27^ and help to explain the simplicity of introducing new drug-binding/protonation sites into MdfA (Fig. 4b), are often overlooked or neglected^1,7^.

These electrostatic interactions, unlike H-bonds, lack stringent geometric requirements but are functionally important for two reasons. Firstly, they mediate substrate-induced proton release (Figs. 3a and 4c). Secondly, they allow the transporter to preferably export substrates carrying positive charge(s), especially if the transporter contains two or more protonatable, acidic amino acids in the multidrug-binding site (such as I239T/G354E). The ability to mediate the substrate-triggered deprotonation is particularly important, since long-range electrostatic interactions may not always contribute to the drug-binding affinity. Indeed, previous studies demonstrated that MdfA and its variant D34N bind a monocationic drug with similar affinity, but only MdfA can transport this drug^11^. In this case, MdfA likely uses D34 to recognize the substrate through long-range charge-charge interactions, which trigger deprotonation rather than enhance binding affinity.

As such, multidrug transporters such as MdfA are polyspecific rather than nonspecific: specificity arises because H-bonds are used to select some drugs and the overall size and shape of substrates need to fit the multidrug-binding site; while promiscuity exists because many structurally dissimilar compounds carrying positive charges are recognized through long-range electrostatic interactions. Thanks to these charge-charge interactions, additional drug-binding and protonation site can be introduced into MdfA if the new acidic amino-acid side-chain is located within the hydrophobic multidrug-binding cavity, and sufficiently far from D34 (Fig. 4b). Importantly, we found that the added amino acid such as G354E is less functionally important than D34, probably because D34 is better suited than G354E for evoking protein conformational changes upon drug-binding and protonation. Notably, the location of protonation site in another MFS multidrug efflux pump LmrP also seems flexible, which can be altered without compromising the transport function^36,37^. The similarity between MdfA and LmrP may reflect the importance and prevalence of long-range electrostatic interactions, which render the binding of cationic drugs particularly flexible in spatial arrangement.

Furthermore, our results offer new clues about the fundamental difference between substrate and inhibitor, implying that potent therapeutics may be designed to evade extrusion by any H^+^-coupled antiporter if they lack the ability to trigger deprotonation, as suggested by our DXC-bound structure (Fig. 7a). Take chloramphenicol for example. In the chloramphenicol-bound Q131R structure, both the C4-OH and C5-OH in chloramphenicol form H-bonding interactions with the side-chain carboxylate of D34, likely to trigger the deprotonation of the transporter^21^. In principle, if both the C4-OH and C5-OH in chloramphenicol are methylated or replaced by hydrogen and/or fluorine atoms, the modified chloramphenicol would be unlikely to deprotonate D34 and hence no longer be exported by multidrug efflux pumps such as MdfA^22,38^. Even though such modification may weaken the interactions between chloramphenicol and the target ribosome^39,40^, this effect may be compensated or offset by further derivatization of chloramphenicol to enhance its binding to the ribosome^41^. Evasion of the transporter-mediated efflux by the modified chloramphenicol, and theoretically any other antimicrobial agent, will likely increase the intracellular concentration of these compounds and improve their efficacy, thereby curbing the emerging crisis of antimicrobial resistance.

## Methods

### Protein expression and purification

The gene encoding MdfA was synthesized (GenScript, NJ) and cloned into a modified pET28b vector with a C-terminal deca-histidine tag. Mutations were introduced into the genes encoding MdfA by the QuikChange method (Agilent Technologies) and were confirmed by DNA sequencing. *E. coli* BL21 (DE3) Δ*acrAB*Δ*macAB*Δ*yojHI* cells^42^ transformed with the expression vectors were grown in LB media to an OD_0.5_ at 600 nm (OD_600_) and induced with 0.5 mM IPTG at 30 °C for 4 h. Cells were harvested by centrifugation and ruptured by multiple passages through a pre-chilled French pressure cell. All the protein purification experiments were performed at 4 °C. Membranes were collected by ultracentrifugation and extracted with 1% (wt/vol) n-dodecyl-β-maltoside (DDM, Anatrace) in 20 mM HEPES-NaOH pH 7.5, 500 mM NaCl, 20% (vol/vol) glycerol and 1 mM tris(2-carboxyethyl)phosphine (TCEP). The soluble fraction was loaded onto Ni-NTA resin in 20 mM Hepes-NaOH pH 7.5, 100 mM NaCl, 25% glycerol, 0.02% DDM and 1 mM TCEP. Protein was eluted using the same buffer supplemented with 500 mM imidazole. The protein sample was promptly desalted and incubated with thrombin overnight. After thrombin cleavage the protein sample was desalted and concentrated to ~20 mg/ml before it was further purified by using gel filtration chromatography (Superdex 200) in 20 mM Tris-HCl pH8.0, 200 mM NaCl, 10% glycerol, 0.2% n-Nonyl-β-Glucoside (Anatrace), 0.025% LDAO (Anatrace), 0.05% DXC and 1 mM TCEP. For biochemical studies of MdfA variants, DDM was used throughout protein purification.

### Protein crystallization and soaking

Prior to crystallization, I239T/G354E was concentrated to ~10 mg/mL and dialyzed extensively against 20 mM Tris-HCl pH 8.0, 200 mM NaCl, 25% (vol/vol) glycerol, 0.2% n-Nonyl-β-Glucoside (NG, Anatrace), 0.025% n-Dodecyl-N,N-Dimethylamine-N-Oxide (LDAO, Anatrace), 0.05% DXC and 1 mM TCEP at 4 °C. Crystallization experiments were performed using the hanging-drop vapor-diffusion method at 22 °C. The protein samples were mixed with equal volume of a crystallization solution containing 100 mM MES-NaOH, pH 6.0, 200 mM NaCl, 100 mM magnesium chloride, 40 mM praseodymium acetate or Pr(OAc)_3_, 30–40% (wt/vol) PEG400. Protein crystals usually appeared within 2 weeks and continued to grow to full size in a month. To soak LDAO or MV into the protein crystals, the I239T/G354E crystals were incubated in a solution containing 100 mM Tris-HCl, pH8.0, 200 mM NaCl, 100 mM magnesium chloride, 60 mM Pr(OAc)_3_, 40% (wt/vol) PEG400, 0.2% NG, 0.05% LDAO or 10 mM MV at 22 °C for 72 h.

### Structure determination and refinement

X-ray diffraction data were collected from the frozen crystals at the beam-lines 23-ID and 22-ID at Argonne National Laboratory. X-ray data were processed using the program suite HKL2000 (ref. ^43^) and further analyzed using the CCP4 package^44^ unless specified otherwise. All the structures were solved by combining molecular replacement and SAD phasing. The Q131R model (PDB 4ZP0) was placed into the unit cell by using the program PHASER^45^. Praseodymium binding sites were identified by difference Fourier analysis and SAD phases were calculated using the program SHARP^46^. The resulting electron density maps were improved by using solvent flattening, histogram matching, cross-crystal averaging and phase extension. Model building was carried out by using the program O^47^. Structure refinement was conducted by using the program REFMAC with experimental phases as restraints^48,49^. The final refined occupancies of the ligands are 1.0 except for that of DXC, which is 0.7. Overall, the diffraction data exhibited moderate completeness, which is likely due to the anisotropic diffraction^50–52^, a low-symmetry space group and the shortage of high-quality crystals. We chose the resolution cutoffs based on the CC_1/2_ (>0.15), data completeness (>40%), and more importantly, whether the inclusion of the high resolution diffraction data improved the quality of the structural models and electron density maps^53,54^.

### Western blot analysis and drug resistance assay

The membrane expression levels of MdfA variants were not affected by the mutations described in this study, as judged by Western blot using an antibody against the His-tag. This observation justifies the comparison of the transport function of MdfA mutants on the basis of the drug resistance assay. For the Western blot, the antibody (Qiagen #34460) was diluted 2500 fold before being mixed with the transfer membranes, and each sample examined on the gel was derived from cell membranes isolated from 80 µg *E. coli* BL21 (DE3) Δ*acrAB*Δ*macAB*Δ*yojHI* cells expressing the MdfA variants. For drug resistance assay, BL21 (DE3) ∆*acrAB*∆*macAB*∆*yojHI* cells expressing the MdfA variants were grown at 37 °C in LB media supplemented with 50 µg/mL kanamycin to an OD_600_ of 1.0. Five consecutive 10-fold dilutions of cells were then prepared (10^−1^−10^−5^), and 4 µL of each dilution was spotted on LB plates (2% agar) supplemented with kanamycin (60 µg/mL), IPTG (0.1–0.2 mM) and various concentrations of the tested compounds. For control experiments, LB plates supplemented with kanamycin (60 µg/mL) and IPTG (0.1–0.2 mM) were used. The ability of the bacteria to form single colonies was recorded after 14 h at 30 °C. The experiments were performed in triplicate and at least three separate transformations were performed for each MdfA variant.

### Fluorescence measurement of H^+^-release

Purified MdfA variants were dialyzed extensively against solutions containing 20% (vol/vol) glycerol, 100 mM NaCl and 0.01% (wt/vol) DDM. For each measurement, protein was diluted to 2 or 4 µM in the same buffer supplemented with 2 µM fluoresceine, a pH-sensitive fluorophore that can be used for quantitative measurement of solution acidity^11,55^. Fluorescence measurement was carried out by using an Olis SLM-8000 spectrofluorometer with excitation and emission wavelengths of 494 nm and 521 nm, respectively. LDAO, MV or DXC was added at the indicated times to reach a concentration of 150 µM, 500 µM, and 2 mM, respectively. Samples (2 mL) were stirred continuously during measurement. Same experiments were repeated three times, which yielded a stoichiometry of 2.0 ± 0.1 H^+^ per I239T/G354E upon the binding of LDAO or MV; 1.0 ± 0.1 H^+^ per MdfA or D34A/I239T/G354E on LDAO, MV or DXC binding.

### Drug-H^+^ antiport assay

Everted (inside out) membrane vesicles were prepared from BL21 (DE3) ∆*acrAB*∆*macAB*∆*yojHI* cells expressing MdfA variants^55,56^. Briefly, cells were grown in LB media to an OD_600_ of 0.5 and induced with 0.5 mM IPTG at 30 °C for 3 h. Cells were harvested by centrifugation and washed once with buffer containing 10 mM Tris-HCl, pH 7.5, 5 mM MgCl_2_, 0.5 mM DTT and 0.25 M sucrose. The cells were disrupted by using a pre-chilled French press and the cell lysate was centrifuged at 10,000 g for 60 min at 4 °C. Membranes were then collected by centrifugation at 100,000×*g* for 60 min at 4 °C. For each measurement, membrane vesicles (100 µg proteins) were added to 2 ml of pre-warmed (30 °C) buffer containing 10 mM Tris-HCl, pH 7.0, 5 mM MgCl_2_ and 1 µM acridine orange^55^. The samples were continuously stirred and fluorescence was monitored with an excitation wavelength of 492 nm and emission wavelength of 525 nm by using an Olis SLM-8000 spectrofluorometer. Prior to addition of substrates, 2 mM lactate was added to energize the membrane and thereby quench acridine orange fluorescence. Upon the addition of 10 µM LDAO, 50 µM MV or 250 µM DXC, fluorescence dequenching occurred due to the extrusion of H^+^ by antiporters that can move drugs into the everted vesicles across membranes. 5 mM NH_4_Cl was finally added to dissipate the H^+^ gradient.

### Reporting summary

Further information on research design is available in the Nature Research Reporting Summary linked to this article.

## Supplementary information

Supplementary Information

Supplementary Data 1

Reporting Summary

Description of Supplementary Data

## Data Availability

Coordinates and structure factors are deposited in the Protein Data Bank under accession codes 6OOM, 6OOP, and 6OOQ. All relevant data supporting the findings of this study are available from the authors upon reasonable request.
