# Supplementary material for: Structure of an engineered multidrug transporter MdfA reveals the molecular basis for substrate recognition
Source: Commun Biol. 2019 Jun 17;2:210. doi: 10.1038/s42003-019-0446-y (PMC6572762; doi:10.1038/s42003-019-0446-y)
Supplement: Supplementary file 1 — Supplementary Information [file 42003_2019_446_MOESM1_ESM.pdf]

Supplementary Information

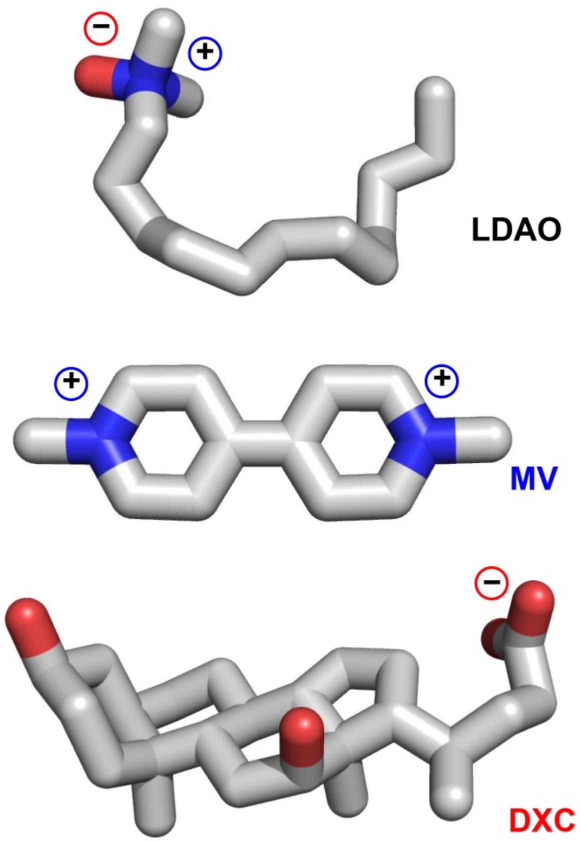

Supplementary Figure 1. Structures and charges of LDAO, MV and DXC. The three compounds are drawn as stick models, atoms that carry positive and negative charges are highlighted by “+” and “-” signs, respectively. The net charges of LDAO, MV and DXC at physiological pH (7.4-7.6) are 0, +2, and -1, respectively.

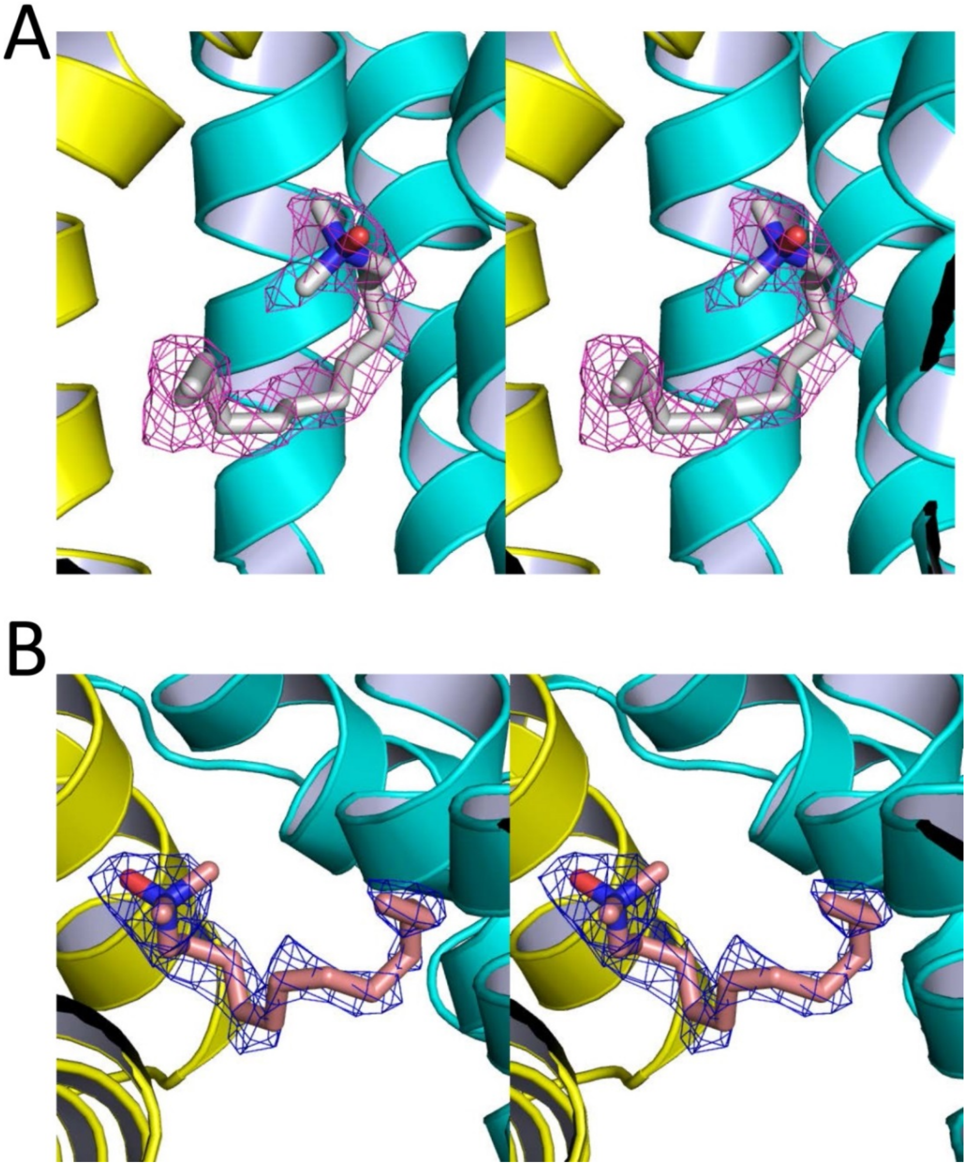

Supplementary Figure 2. Stereo view of the bound LDAO molecules in I239T/G354E. The experimental electron density map (magenta or blue mesh, 1.5σ) was calculated to 2.2 Å resolution by using the density-modified SAD phases and overlaid onto the final models of LDAO1 and LDAO2. Density modification included solvent flattening, histogram matching, cross-crystal averaging and phase extension. I239T/G354E is shown in ribbon representation and LDAO molecules are drawn as sticks. The N and C domains of I239T/G354E are colored cyan and yellow respectively. LDAO1 and LDAO2 are colored grey and light pink, respectively.

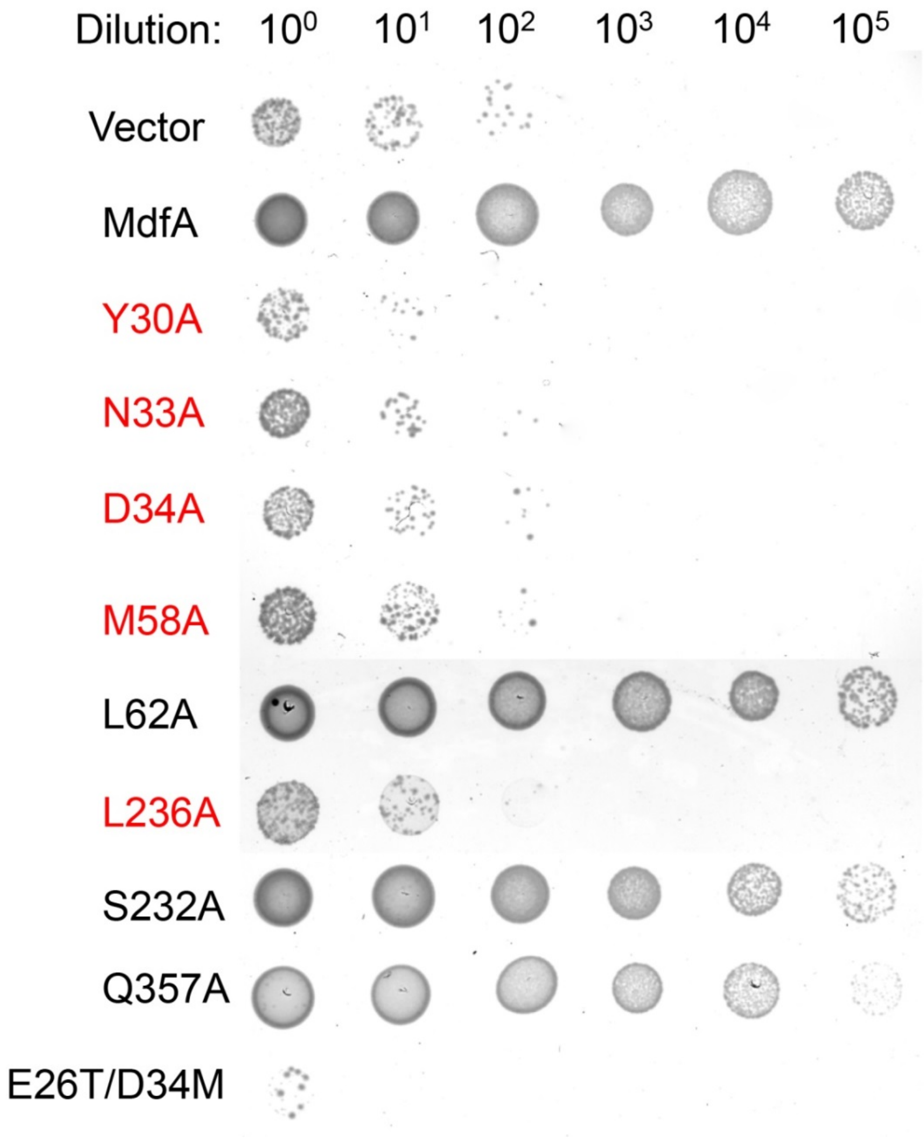

Supplementary Figure 3. Functional importance of the LDAO-binding site in MdfA. Growth of E. coli expressing MdfA variants on solid media supplemented with kanamycin, IPTG and 0.005% LDAO. Bacterial growth on the control plates supplemented with only kanamycin and IPTG gave the same results for all MdfA variants. Five consecutive 10-fold dilutions of bacteria were plated from left to right and incubated overnight. The experiments were repeated >3 times and the representative results are shown. Similar observations were made when the growth media was supplemented with 0.01% LDAO.

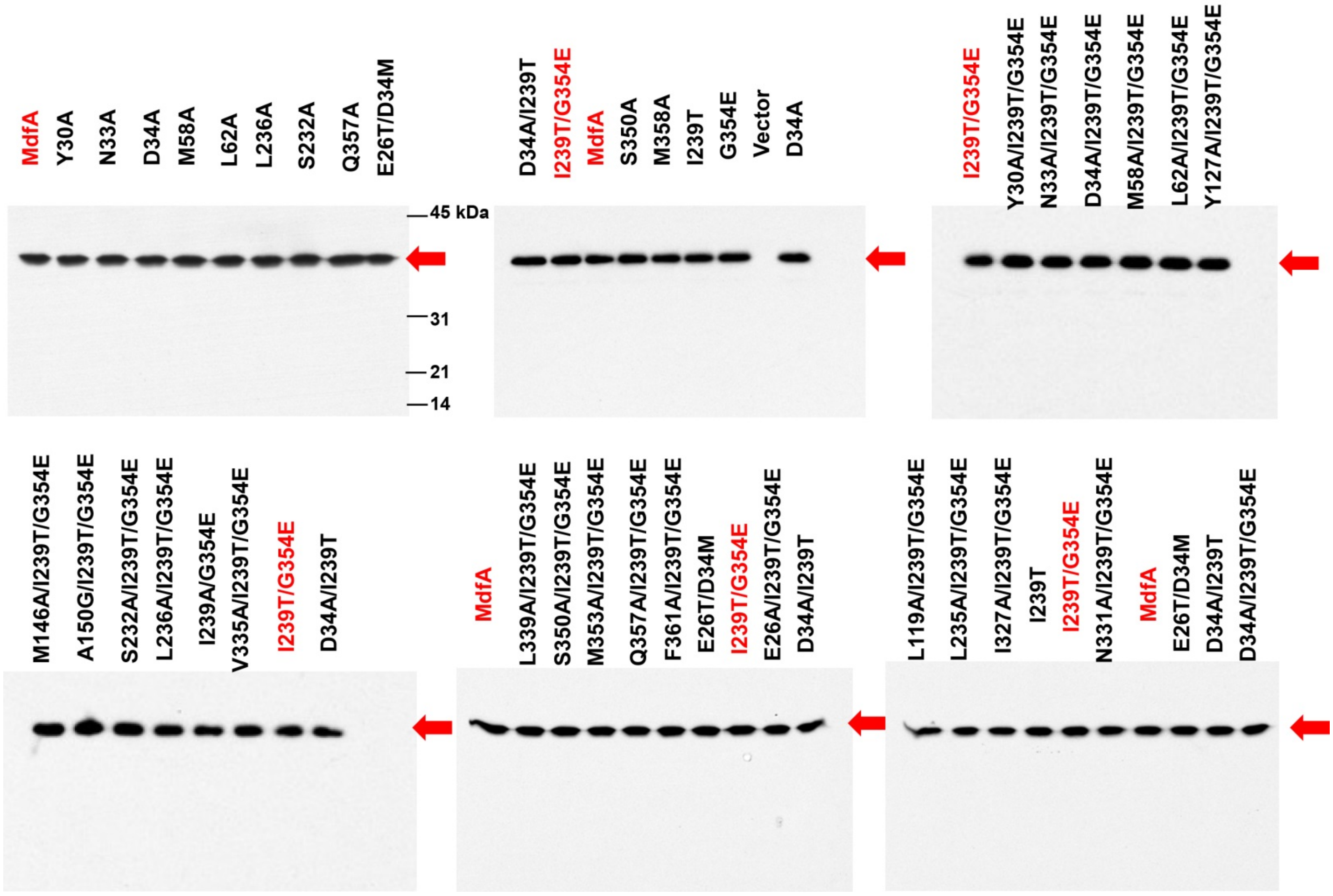

Supplementary Figure 4. Western blot analysis of MdfA variants examined in this study.

Western blot analysis of MdfA variants in membrane preparations, which was performed by using an antibody against the His-tag. This analysis suggested that the MdfA variants were expressed at similar levels. Positions of molecular weight markers are indicated and the bands that correspond to the MdfA variants are highlighted by red arrows.

I239T/G354E

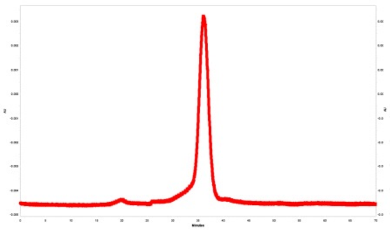

Y30A/I239T/G354E

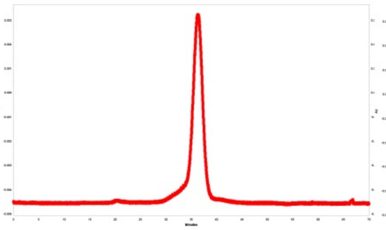

D34A/I239T/G354E

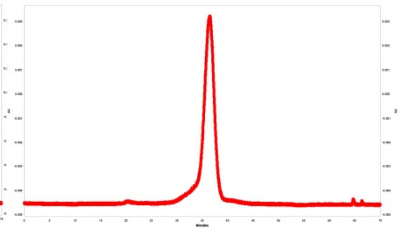

M58A/I239T/G354E

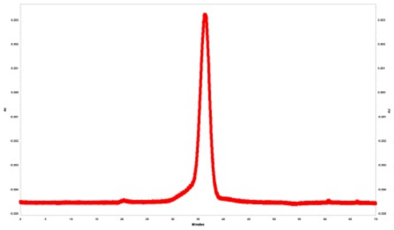

L62A/I239T/G354E

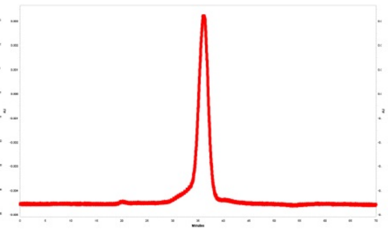

Y127A/I239T/G354E

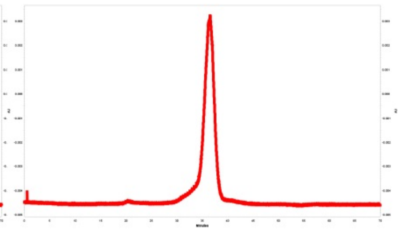

L236A/I239T/G354E

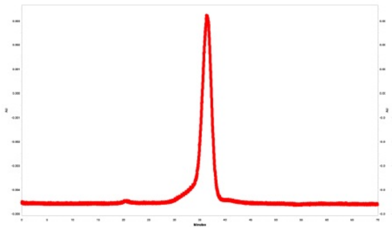

V335/I239T/G354E

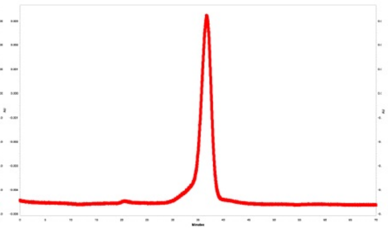

L339A/I239T/G354E

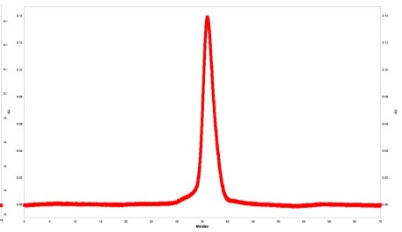

F361A/I239T/G354E

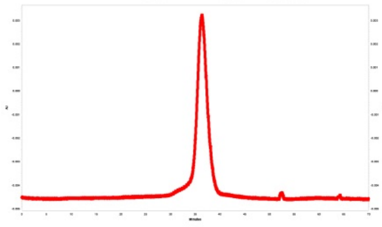

E26T/D34M

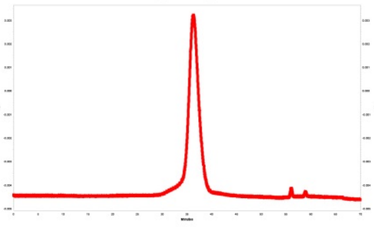

MdfA

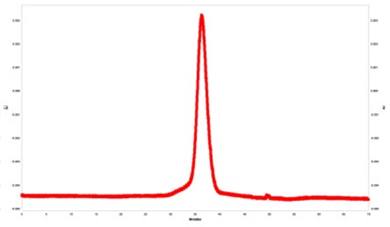

D34A/I239T

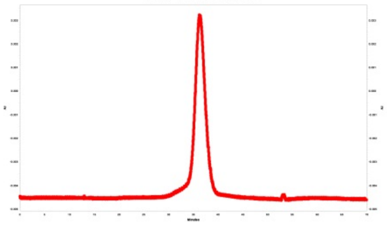

E26A/I239T/G354E

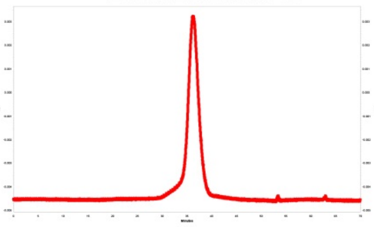

L119A/I239T/G354E

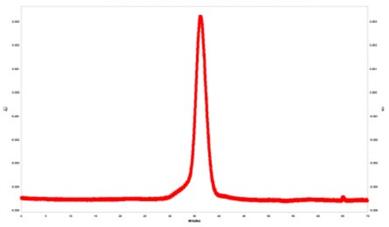

L235A/I239T/G354E

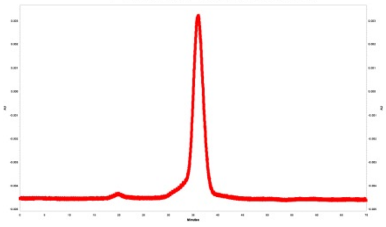

I327A/I239T/G354E

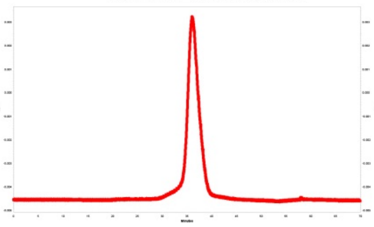

Supplementary Figure 5. Analytical gel filtration chromatograms of relevant MdfA variants. For each variant, ~0.2 gram of bacterial membrane was used for purification using the detergent DDM. In each chromatogram, the UV absorption at 280 nm (0-0.16) was plotted against the elution time (0-70 min). These detergent-purified MdfA variants all eluted at ~14ml as sharp, symmetrical peaks, strongly suggesting that these variants are well-folded. For each variant, ~200 µg of purified protein was loaded onto a Superdex 200 column (~24 ml) pre-equilibrated in 20 mM Tris-HCl pH 8.0, 100 mM NaCl, 10% glycerol, 0.02% DDM and 0.5 mM TCEP, with a flow rate of 0.4 ml/min. All experiments were conducted at 4°C. These size exclusion chromatography results are consistent with our Western blot analysis (Supplementary Fig. 4), suggesting that the tested MdfA variants were all expressed at similar levels in *E. coli*.

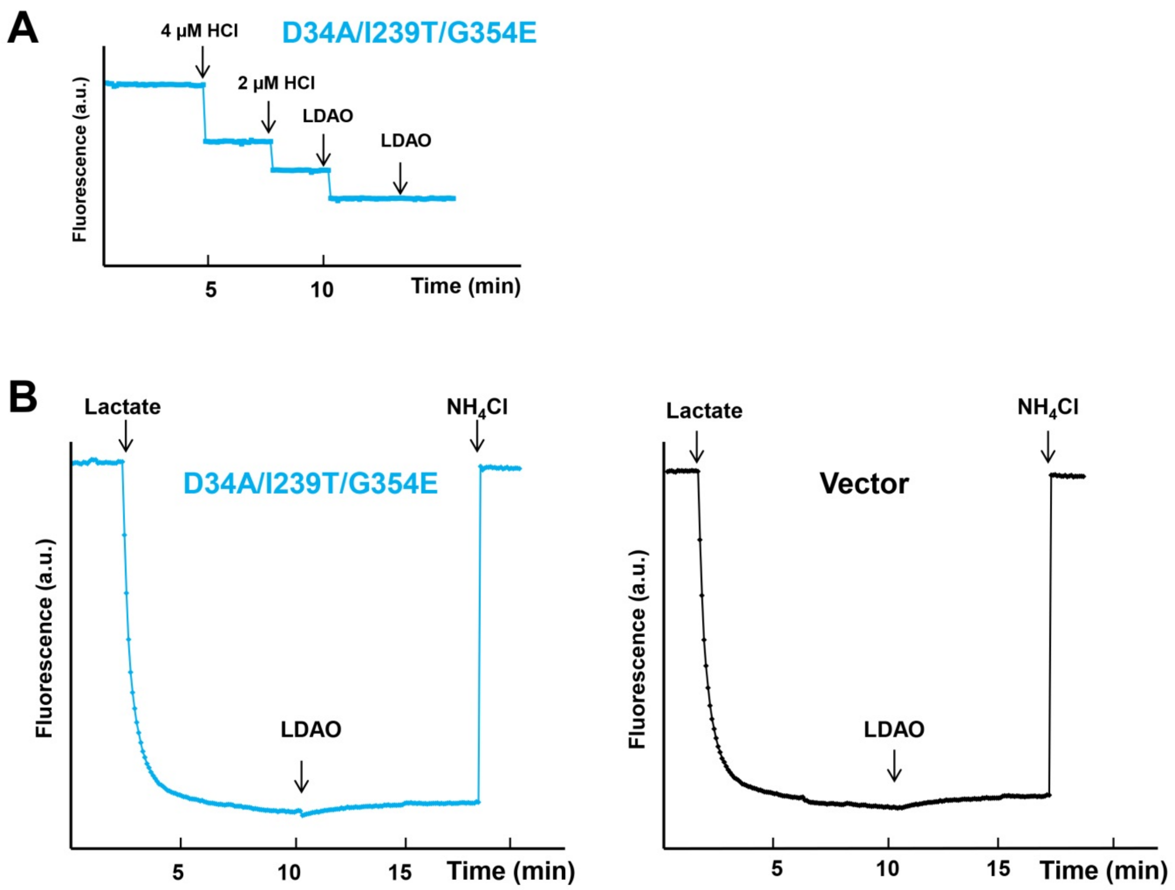

Supplementary Figure 6. Functional characterization of D34A/I239T/G354E. (**A**) Fluorescence measurement of a solution containing 2 µM D34A/I239T/G354E, revealing its ability to release one proton per protein molecule upon LDAO binding. Further addition of LDAO caused no more reduction of fluorescence, suggesting that the binding of LDAO to D34A/I239T/G354E was saturated. (**B**) LDAO/H^+ antiport examined in the everted (inside out) membrane vesicles expressing D34A/I239T/G354E (left panel) and vector (right panel). H^+ movement was monitored by measurement of acridine orange fluorescence, which is shown in arbitrary units (a.u.). LDAO failed to trigger the counter-movement of H^+ in the inside out membrane vesicles harboring D34A/I239T/G354E or vector. The traces are representative of experiments performed in triplicate using two different preparations of everted membrane vesicles.

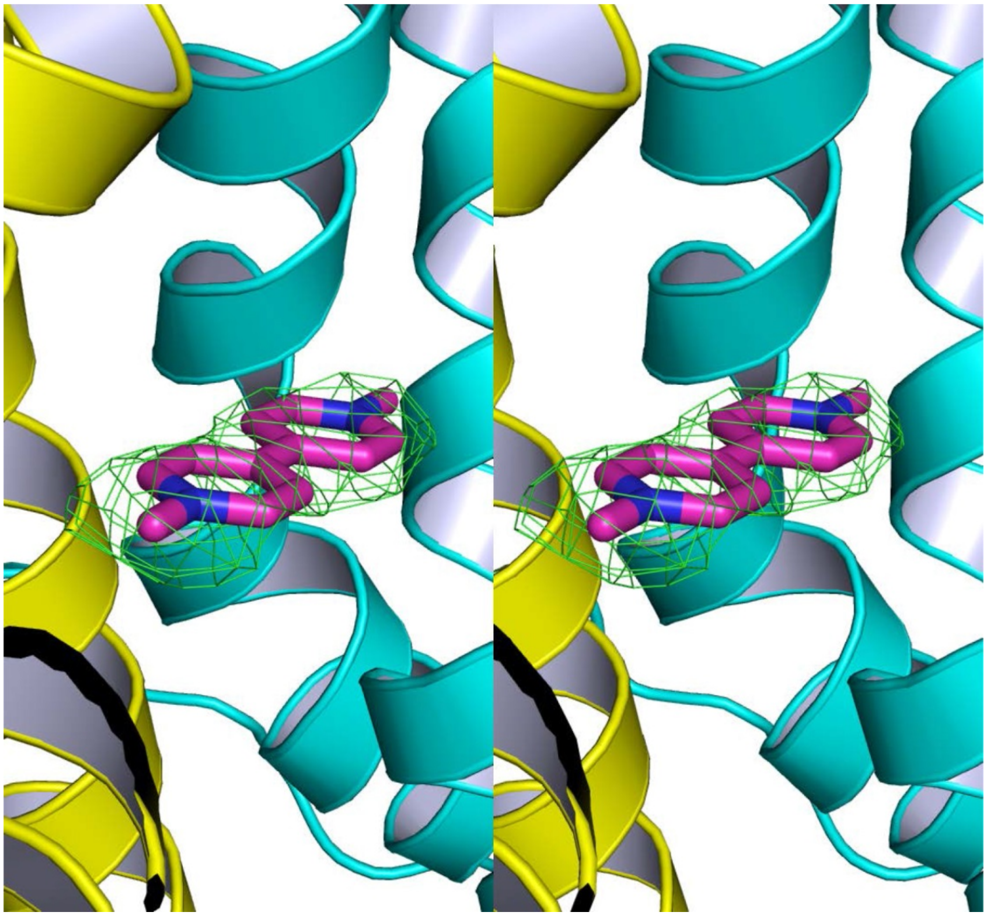

Supplementary Figure 7. Stereo view of the bound MV in I239T/G354E. The experimental electron density map (green mesh, 1.5σ) was calculated to 2.8 Å resolution by using the density-modified SAD phases and overlaid onto the final model of MV. I239T/G354E is shown in ribbon representation and MV is drawn as sticks. The N and C domains of I239T/G354E are colored cyan and yellow respectively. MV is colored magenta.

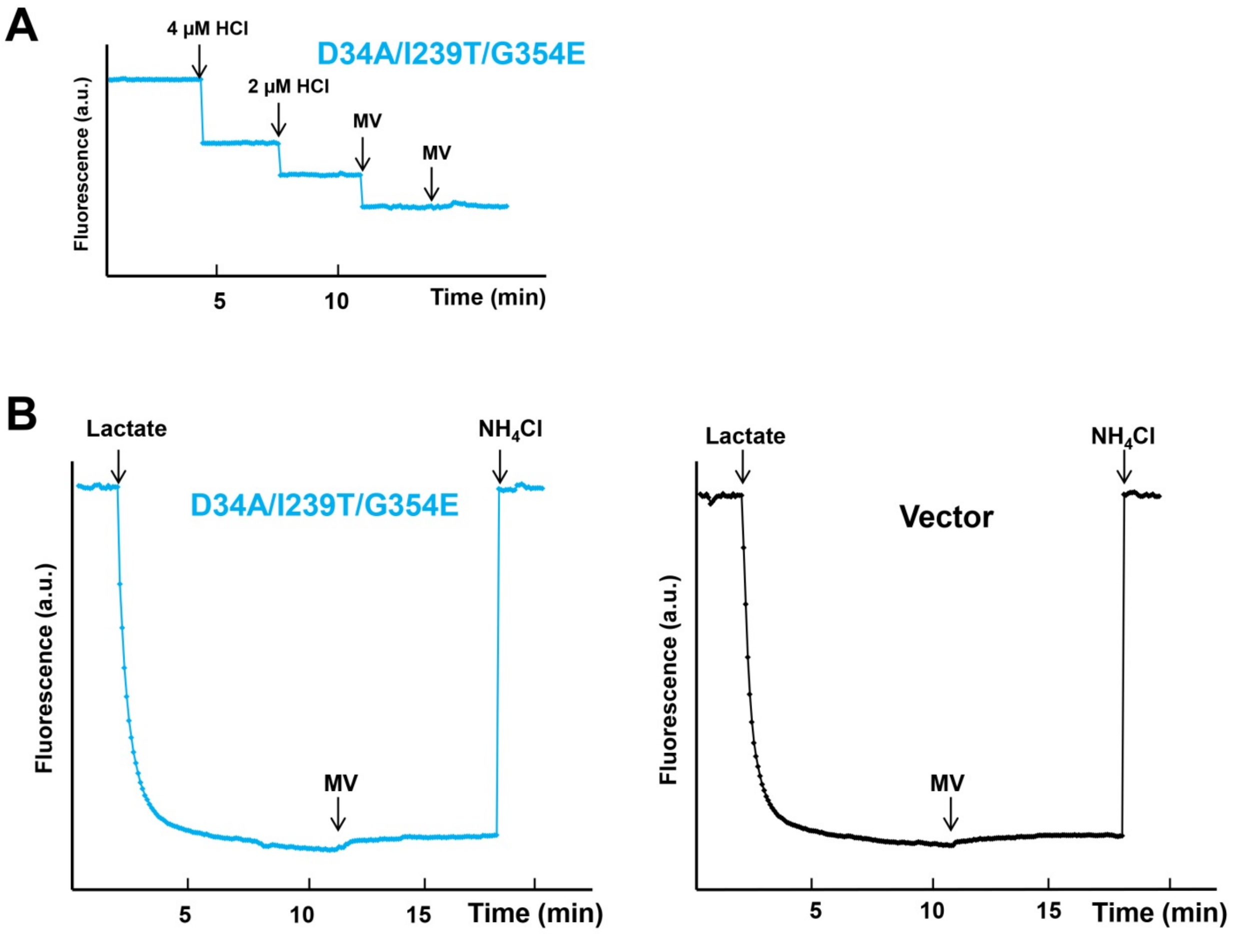

Supplementary Figure 8. Functional characterization of D34A/I239T/G354E. (**A**) Fluorescence measurement of a solution containing 2 µM I239T/G354E, revealing its ability to release one proton per protein molecule upon MV binding. (**B**) MV/H^+ antiport examined in the everted membrane vesicles expressing I239T/G354E (left panel) and vector (right panel). H^+ movement was monitored by measurement of acridine orange fluorescence, which is shown in arbitrary units (a.u.). By contrast, MV failed to trigger H^+ movement in the everted membrane vesicles harboring D34A/I239T/G354E or vector. The traces are representative of experiments performed in triplicate using two different preparations of everted membrane vesicles.

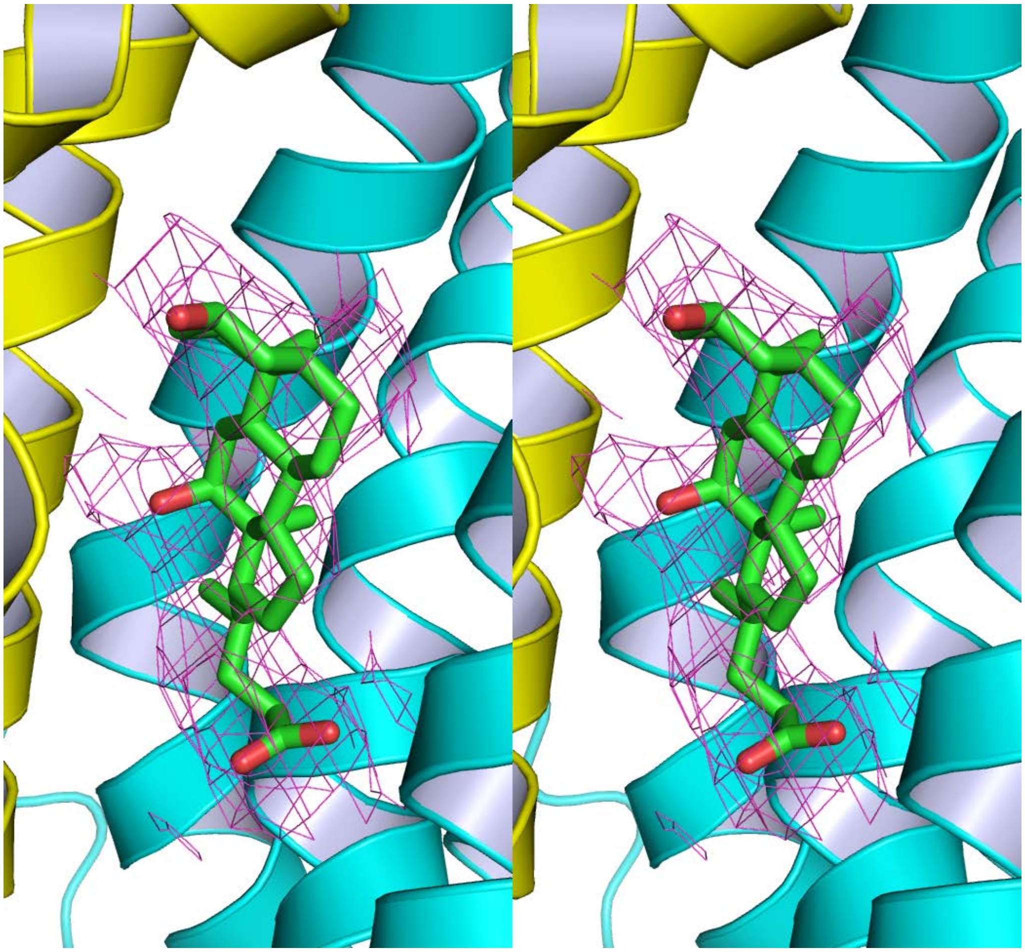

Supplementary Figure 9. Stereo view of the bound DXC in I239T/G354E. The experimental electron density map (magenta mesh, 1.0σ) was calculated to 3.0 Å resolution by using the density-modified SAD phases and overlaid onto the final model of DXC. I239T/G354E is shown in ribbon representation and DXC is drawn as sticks. The N and C domains of I239T/G354E are colored cyan and yellow respectively. MV is colored green.

A

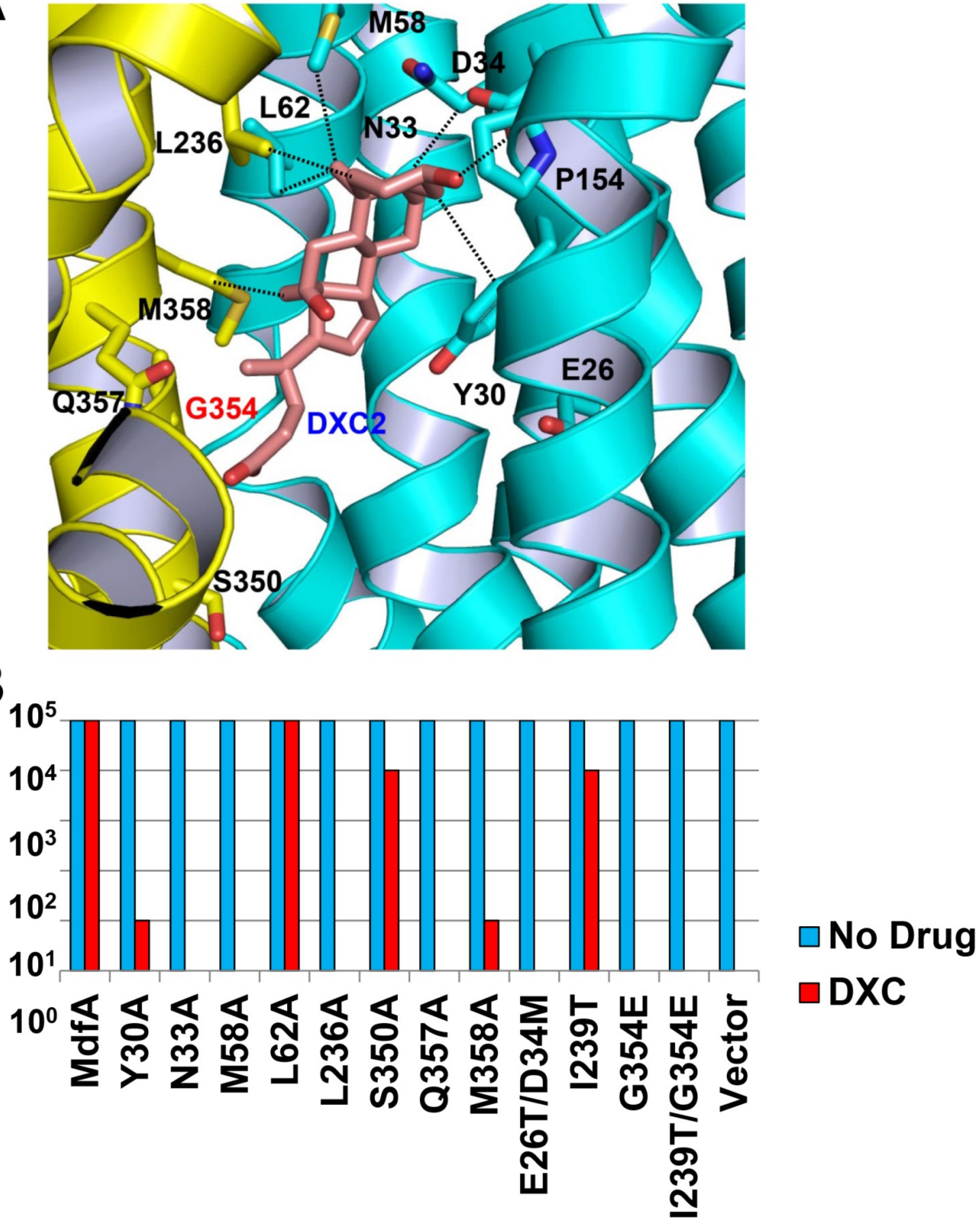
M58
D34
L62
N33
L236
P154
M358
E26
Y30
Q357
G354
DXC2
S350
10^5
10^4
10^3
10^2
10^1
10^0
MdfA
Y30A
N33A
M58A
L62A
L236A
S350A
Q357A
M358A
E26T/D34M
I239T
G354E
I239T/G354E
Vector
No Drug
DXC

B

Supplementary Figure 10. Functional importance of the DXC-binding site in MdfA. (**A**) Close-up view of the DXC-binding site in Q131R (PDB 4ZP0). Protein is shown in ribbon representation and the bound DXC is drawn as sticks and labeled DXC2. The N and C domains of Q131R are colored cyan and yellow, respectively. DXC2 is colored light pink. Close-range interactions are shown as dashed lines. Notably, E26, P154, S350, G354, or Q357 makes no direct contact with DXC2. (**B**) Bacteria expressing the I239T/G354E variants were tested for DXC resistance in solid media. Five consecutive 10-fold dilutions of bacteria were prepared, and 4 µl of each dilution were plated on plates containing kanamycin, IPTG, 500 µg/ml DXC. The ability of bacteria to form single colonies was visualized after overnight incubation. The height of the bars corresponds to the maximal dilution at which bacterial growth was observed. The experiments were repeated >3 times.

Supplementary Table 1

Distances between the bound ligands and relevant amino acids in I239T/G354E.

I239T/G354E	LDAO	MV	DXC
E26		7.9 Å	
Y30	4.0 Å		
N33	4.0 Å		
D34	3.7 Å	7.3 Å	
M58	3.7 Å		
L62	3.8 Å		
L119		3.8 Å	
Y127	3.9 Å		
M146	3.9 Å		
A150	3.9 Å		3.7 Å
S232	4.0 Å	3.7 Å	3.9 Å
L235		4.0 Å	3.7 Å
L236	4.0 Å	4.0 Å	4.0 Å
I239T	3.9 Å		
I327		3.9 Å	
N331			2.7 Å
V335	3.8 Å		
L339	3.9 Å		
S350	4.0 Å		
M353	3.9 Å		
G354E	3.7 Å	5.2 Å	
Q357	3.0 Å		
F361	4.0 Å	4.0 Å	
