# Supplementary material for: Structure of an engineered multidrug transporter MdfA reveals the molecular basis for substrate recognition
Source: Commun Biol. 2019 Jun 17;2:210. doi: 10.1038/s42003-019-0446-y (PMC6572762; doi:10.1038/s42003-019-0446-y)
Supplement: Supplementary file 4 — Description of Supplementary Data [file 42003_2019_446_MOESM4_ESM.pdf]

Description of Additional Supplementary Files

File Name: Supplementary Data 1
Description: Source data for figures
